# State-of-the-Art Review of Artificial Neural Networks to Predict, Characterize and Optimize Pharmaceutical Formulation

**DOI:** 10.3390/pharmaceutics14010183

**Published:** 2022-01-13

**Authors:** Shan Wang, Jinwei Di, Dan Wang, Xudong Dai, Yabing Hua, Xiang Gao, Aiping Zheng, Jing Gao

**Affiliations:** 1 State Key Laboratory of Toxicology and Medical Countermeasures, Beijing Institute of Pharmacology and Toxicology, Beijing 100850, China; WSSS980503@163.com (S.W.); dijinwei1996@163.com (J.D.); wdgs223@163.com (D.W.); dxddai@163.com (X.D.); huayabing1111@xzhmu.edu.cn (Y.H.); 2 Key Laboratory of Natural Medicines of the Changbai Mountain, College of Pharmacy, Yanbian University, Yanji 133002, China; 3 Department of Pharmacy, Xuzhou Medical University, Xuzhou 221004, China

**Keywords:** artificial neural networks, artificial intelligence, pharmaceutical formulation, multilayer perceptron

## Abstract

During the development of a pharmaceutical formulation, a powerful tool is needed to extract the key points from the complicated process parameters and material attributes. Artificial neural networks (ANNs), a promising and more flexible modeling technique, can address real intricate questions in a high parallelism and distributed pattern in the manner of biological neural networks. The data mined and analyzing based on ANNs have the ability to replace hundreds of trial and error experiments. ANNs have been used for data analysis by pharmaceutics researchers since the 1990s and it has now become a research method in pharmaceutical science. This review focuses on the latest application progress of ANNs in the prediction, characterization and optimization of pharmaceutical formulation to provide a reference for the further interdisciplinary study of pharmaceutics and ANNs.

## 1. Introduction

The development of artificial intelligence (AI) undoubtedly has revolutionary significance since AI uses big data as a more effective human substitute. To be factual, AI is a field focused on intellectual tasks that are generally performed by humans [1]. One subfield of AI is called machine learning (ML) and its research content is the learning methods suitable for machines, which allow machines to learn useful knowledge from external input information, as humans do, and to improve their processing ability on the basis of newly acquired information. This means that ML can keep learning to improve analytical accuracy and make judgments on and predictions for problems by constructing algorithms that can be mastered independently [2,3]. Deep learning (DL) is one of the methods of machine learning, which enables computer systems to be improved by experience and data. In most cases, DL uses artificial neural networks (ANNs) and multilayer nonlinear processing units for handling a vast amount of experimental data [4,5].

**Figure 1:**
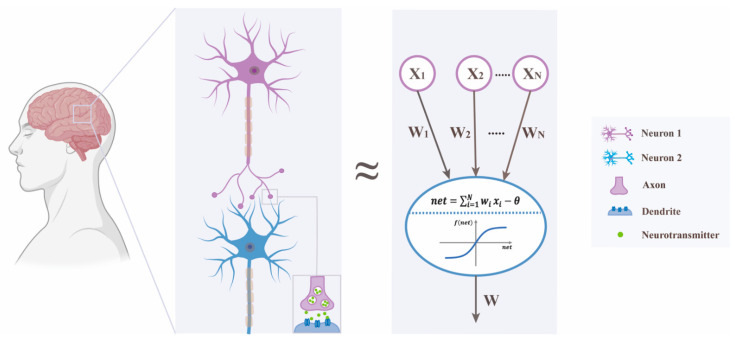
The actual neural network of the human brain and an ANN. In the brain’s central nervous system, a neuron receives external stimulus by dendrites and transmits processed signals along the axon to the axon end—synapses, and then releases neurotransmitters. The neurotransmitters diffuse through the synaptic gap and emit excitatory or inhibitory electrical signals to receptor neurons, according to the synapse type. The strength of a synapse (weight) can be regulated by the transmitted signals; thus, the synapses can begin to learn. The same step is shown in the work of ANNs. A neuron takes the output of other neurons as its input and then performs a weighted summation of these inputs. If the sum is greater than its threshold (θ), the neuron is in an excited state and has the output of “1”, otherwise the output of “0” shows that the neuron is in an inhibited state [9].

The design of ANNs simulates the structure of the actual neural networks of the brain’s central nervous system to a certain extent [6], so ANNs can recognize, classify, learn and acquire skills based on experience (Figure 1). An ANN consists of a number of connected neurons. Each neuron acts as a parallel distributed processor, which can perform large-scale computations for data processing and classification [7]. Compared to computers that are clearly separated from calculation and storage, the calculation and storage of brains are highly integrated in the connections of neurons (nerve cells) and their synapses. In 1958, the concept and model of perceptron was first proposed by Frank Rosenblatt: a network composed of the input layer and the output layer became the first machine that could learn weights based on each sample, which formed the basis of ANNs [8,9,10]. The next key milestone in ANN development was John Hopfield introducing the nonlinearity between input and output data in 1982, thus a novel method was explained. This innovative perceptron structure made remarkable progress in ANN running results [11,12]. Later, Rumelhart et al. re-studied and invented the error backpropagation (BP) algorithm [13,14]. With the progress of the Hopfield network and the success and spread of the BP algorithm training multilayer perceptron (MLP), the research of artificial neural networks gained popularity and reached its peak in the early 1990s. In recent years, DL has generated a new upsurge, which vigorously promotes the development of artificial intelligence.

**Figure 2:**
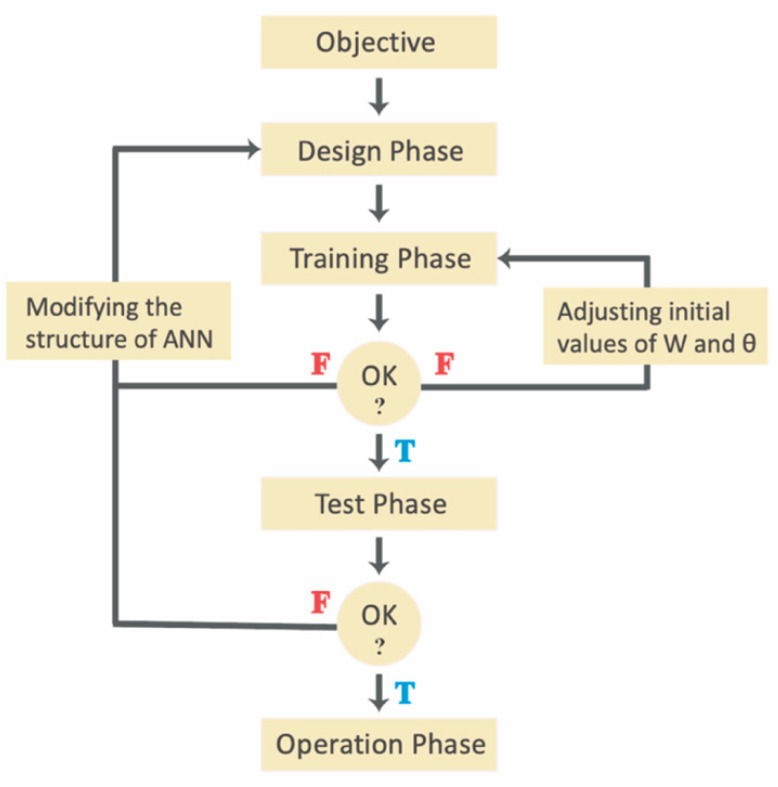
The design and implementation of an ANN.

ANNs, a machine learning technology, have a similar learning pattern. Their architecture is changed according to the information provided during the learning phase, i.e., an ANN learns from past experiences and mistakes to achieve target values, in the manner of mammalian neurons [11]. Consequently, the probability of system errors decreases. As an automatic knowledge extraction tool, an ANN is not limited to some fixed mathematical formulae [15]. Meanwhile, ANNs do not need exact knowledge of the input information in terms of network building. The establishment process of an ANN is depicted in Figure 2. Remarkably, the structure of an ANN is inspired by neuroscience (biological neural network), but it does not directly replicate the working system of biological neurons [16]. ANNs draw inspiration from many fields, especially statistics, applied mathematics, computing science, pattern recognition, etc. [6,10].

The progress of artificial intelligence has brought a positive impact on pharmaceutical formulation development as well. The field of pharmaceutics has the characteristics of strong practicality and application comprehensiveness. It is a technology that studies the basic theory, prescription design (main drug and excipients), preparation process, quality control (quality difference, content, dissolution, etc.) and rational application of drug dosage forms. The research and development of drug formulations demand huge amounts of time and cost, which spurs us on to apply more innovative and interdisciplinary strategies and technologies. Integrating multiple research perspectives in different disciplines can produce more novel, efficient and systematic solutions. Hussain et al. put forward the idea of introducing artificial neural network in pharmaceutical science for the first time in 1991 [17,18]. Compared to traditional methods, ANNs have the characteristics of self-adaptability, strong fault tolerance, high-speed parallelism, few application restrictions, etc. [6]. In addition, a main superiority of ANNs is that they can combine literature-based data with experimental data to solve problems. These superior abilities encourage their implementation in aspects of pharmaceutical science, which makes ANNs a powerful tool to deal with many research issues [19].

So far, many researchers have introduced the work of artificial neural networks into the modern pharmaceutical industry, especially for dealing with data and the vague relationships often faced in the process of research and development. This article introduces and summarizes the structures of ANN models and discusses the application advances of ANNs in the prediction, characterization and optimization of pharmaceutical formulation.

## 2. Models of Artificial Neural Networks

The applicability of ANNs can be enhanced by combining distinct model structures and optimization algorithms. Hence, the learning performance of ANNs depends on the network structures, to a large extent [20]. Artificial neural network models can be divided into many types according to the mutual combination relationship between neurons. Some of the well-known ANN models include: the multilayer perceptron (MLP); the radial basis function neural network (RBFNN); the convolutional neural network (CNN); the Kohonen network; and the recurrent neural network (RNN) [21,22]. ANN models with distinct structures have been developed and applied to deal with various problems.

For an image, the number of learning parameters is usually more than imagined. The CNN is the most commonly used supervised learning technique for images and has made many breakthroughs in image recognition and computer vision. Its typical application is to recognize handwritten characters, authentic color photographs, etc. The introduction of CNNs in many other fields should also be proposed, including object detection, scene labeling, visual saliency detection and pose estimation [23].

Kohonen networks with an unsupervised learning mode are also called self-organizing map (SOM) neural networks. A SOM is composed of the input layer and competition layer (the output layer), and it uses competition learning to classify data. The basic concept is that each neuron in the competition layer competes to respond the input data and one neuron ultimately wins, which represents the classification of input patterns. Hence, SOMs are widely used in many information processing fields, such as speech recognition, classification, clustering, etc.

RNNs are often recommended in voice recognition, language translation, natural language understanding and music synthesis, on account of it being suitable for handling time-series data. That means that the output of a neuron is not only related to the current input but is also related to the output of certain neurons at a previous time or several previous moments. Consequently, an RNN is a network with “memory” and that “memory” plays a pivotal role in the modeling of sequential data.

On one hand, the MLP is the basis of ANN modeling and brings ANN universality. After understanding MLPs thoroughly, the whole framework of artificial neural networks will be easily connected. On the other hand, most ANN models introduced to pharmaceutical science are static neural networks, especially MLPs and GRNNs (generalized regression neural networks, an improved kind of RBFNN). Hence, starting from the MLP, the components and elements of the MLP and GRNN models are introduced in detail.

### 2.1. Multilayer Perceptron and Backpropagation Neural Network

**Figure 3:**
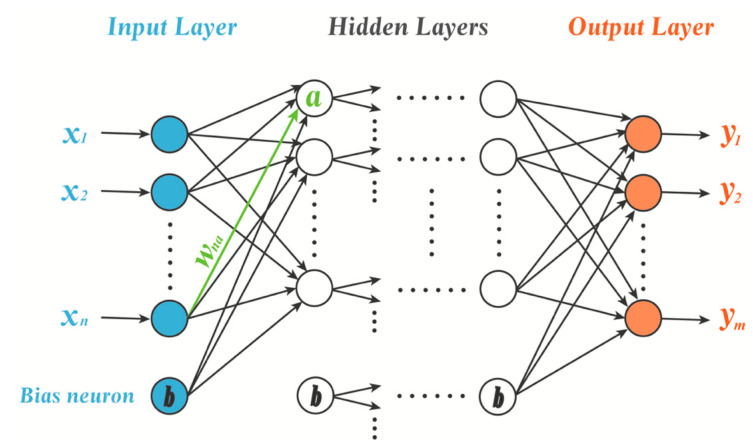
The essential framework of a multilayer perceptron (MLP).

As it is a typical feedforward ANN model, the multilayer perceptron contains an input layer, several hidden layers and an output layer (Figure 3). From a mathematical point of view, an MLP can be considered to express a certain mapping relationship between elements. Among the many ANN architectures, the MLP is one of the most widely used and computationally powerful ANN models in pharmaceutical science [24].

The universal approximation theorem has proven that, for an MLP, if the number of hidden layer neurons and the form of activation functions are not limited, then $y_{k}=\underset{k}{\int }\left(x_{1},x_{2},\ldots ,x_{N}\right)$ can approximately represent any n-variate function [25,26,27]. However, it cannot ensure that the MLP has the ability to learn knowledge and generalize normally [10]. Additionally, “feedforward” does not mean that signals in the network cannot be propagated backward, but that the network topology has no loop [28].

Another major concept is the backpropagation (BP) algorithm. It is a parameter update type of supervised learning, which is easy to use and has a high level of accuracy. Since the 1980s, the BP algorithm has been the core training method of ANNs [22]. The training of the BP algorithm includes two steps: the forward propagation of signals and the backpropagation of the error gradient [20,27], i.e., the result is calculated from the input to the output direction and the error signal of the sample is also obtained. When the weights and thresholds are adjusted, the error is transmitted from the output to the input direction and is distributed to all the neurons in each layer. Each layer of the MLP will calculate the gradient using the error information from the backward direction, and they will use the gradient descent (GD) principle to update the weight of each neuron [27]. Additionally, the backward propagation has the same computational complexity as the forward propagation. The limitation of the BP algorithm is that it easily falls into a local minimum.

A concept that needs to be distinguished is that the BP algorithm refers to the method used to obtain the parameter gradient of the error of each layer in the network, and the GD method utilizes that gradient for learning. In detail, the gradient of the training error of each neuron parameter is calculated and moved forward, layer by layer, from the output layer. Then, according to the principle of gradient descent, the weights are adjusted and updated appropriately along the negative gradient direction. A multilayer feedforward neural network trained by the BP algorithm is called a BP neural network (BPNN) [28]. The training of MLPs generally uses the BP algorithm to modify the weights and thresholds [29], so the two concepts can refer to the same network in most cases.

Madzarevic et al. [30] optimized and predicted the ibuprofen release from three-dimensional (3D) printed tablets using two ANNs, which were modelled by different software (STATISTICA and MATLAB). A supervised MLP and the BP algorithm were used for modeling ANN 1 (3-8-5) and ANN 2 (4-5-5-6-5-6-4). The R^2^ of the experimental value and predicted value were 0.9811 (ANN 1) and 0.9960 (ANN 2). In this study, MLPs can offer a guide to ibuprofen dissolution behavior effectively, based on the excipients and process parameters.

### 2.2. Generalized Regression Neural Network

A GRNN, an ANN using the radial basis function as the activation function, has a strong nonlinear mapping capacity and possesses additional advantages over RBFNNs in some respects [31]. The structure of GRNNs is much clearer because they have fewer layers, which also attributes the significant advantage of a short running time to GRNNs [21].

**Figure 4:**
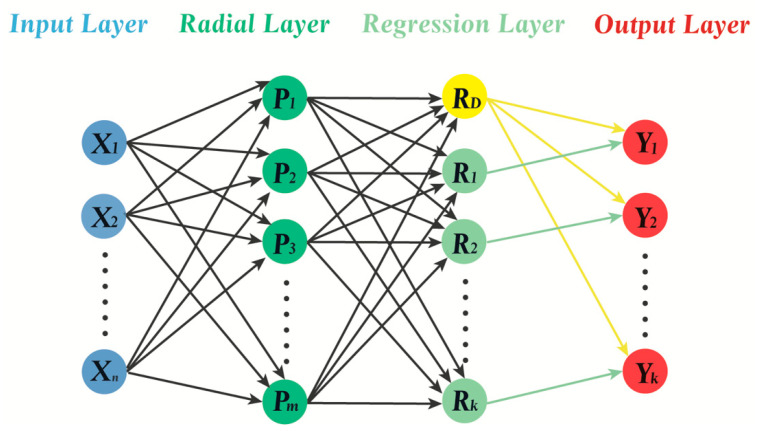
The structure of a GRNN. The radial layer nodes must match the number of training samples (m) and the number of neurons in the regression layer (k + 1) is equal to that in the output layer (k) plus one.

Generalized regression neural networks have four layers: the input layer; the radial layer; the regression layer; and the output layer (Figure 4) [22]. The training algorithm of the radial layer is a clustering algorithm, such as subsampling, k-means and Kohonen training [32]. A GRNN models its function according to the training data directly, without proposing a network structure in advance. Generally, a GRNN is trained by a two-step method: the center of the neuron is determined and then the parameters are defined.

The difference between a GRNN and classic neural networks is that each weight of the GRNN is represented by the weight distribution, which allows more parameter combinations to be considered at the same time [32]. Since the network converges to an optimized regression with many samples, the prediction effect is also better when the sample size is small. GRNNs have significant advantages in actually classifying and fitting, especially when the accuracy of the data to be analyzed is relatively poor [31].

GRNNs are involved in many aspects of pharmaceutical science research [32,33,34,35,36,37]. It is possible to predict drug release based on GRNNs. In a study by Stanojević et al. [38], they predicted the dissolution curve of 3D printed atomoxetine tablets using two types of ANN. A self-organizing map was used to visualize the effect of the inputs on atomoxetine release and a GRNN was then used to predict the atomoxetine release. The training algorithm of the radial layer was k-means. The atomoxetine loading and tablet thickness were the independent variables and the released amounts at 6 time points (0.25, 0.5, 1, 2, 4 and 6 h) were the dependent variables for both networks. The result showed that the predicted dissolution profiles for the two testing formulations were similar to experimental data, with a similarity factor ($f_{2}$) of 51.05 and 70.13, respectively. Thus, the GRNN established in this study had an acceptable prediction ability for the drug’s dissolution behavior.

The application conditions for each type of ANN are different, so we are able to select the best one by testing several models. Behzadi et al. [39] compared the training abilities and the accuracies of predicting product performance by fluid bed granulation process parameters of two types of ANN: an MLP and a GRNN. The result illustrated that the overall prediction accuracy by the GRNN was better than the MLP. The GRNN showed a stronger capacity to verify the granulation processes.

## 3. Progress of Applications of ANNs in Pharmaceutical Science

The superiority of ANNs in handling noisy and nonlinear data allows it to be suitable for dealing with many formulation tasks [33,40]. When constructing a model, the ANN need not have an in-depth knowledge of the input data and to only draw support from a lot of training in order to modify the weights in the network. At the same time, one of the most valuable characteristics of an ANN is its generalization capability, which means that once trained, the ANN can play the role of predicting outputs from new datasets. Hence, ANNs have the key feature of automatic feature extraction, which can be applied to solve complex realistic problems in statistics and model prediction.

A variety of ANN functions can be generalized for classification, prediction and modeling [41]. Based on these abilities, the applications of ANNs in pharmaceutical science can be summarized as the prediction, characterization and optimization of pharmaceutical formulation, as shown in Table 1.

### 3.1. Prediction of Drug Release Behavior In Vitro

ANNs reflect an uncomplicated and dependable system that may tackle varied issues related to data analysis and behavior prediction [81]. Release/dissolution behavior is the core of drug development and the focus of pharmaceutical researchers. In general, most of pharmaceutical formulation release models are founded on Fick’s second law of diffusion. On account of all data being regarded as equal weight, these conventional approaches have some limitations [82]. Meanwhile, complex relationships exist between formulation and processing variables, and the drug release behaviors make it difficult to describe release phenomenon using computationally simple empirical models, including the levels of active pharmaceutical ingredient (API) and excipient(s), the possible interactions between the drug(s) and excipient(s) and the influences of many processing operations on drug release profiles [83]. For example, leuprolide (cationic peptide), the API of leuprolide acetate sustained release microspheres, has the potential to interact with the negatively charged free carboxyl end-group of PLGA at neutral or low pH [84,85], which may greatly impact the release behavior and storage stability.

On one hand, selecting an appropriate formulation with various compositions to obtain the required release/dissolution behavior is a vastly laborious method of developing pharmaceutical products. Based on a great number of tests and the experiences of researchers, a familiar way is to carry out a series of physicochemical methods to obtain a better profile. On the other hand, especially for sustained release dosage forms, such as nanoparticles, microspheres and liposomes, the property of long-term release under conditions simulating body temperature in vitro is inconvenient for the necessary testing in each period. Although accelerated release tests in vitro are often used in labs to obtain the release behaviors of sustained release dosage forms, this strategy produces variant release profiles due to the different methods and may produce some undesirable results attributed to the severe conditions (high temperature, low pH, etc.), for example, profile fragments are difficult to distinguish or are missing and release mechanisms are changed at various release phases.

Therefore, tools used to assist the prediction of the release profiles of drugs that are high-speed and effective are very feasible. For instance, developing an ANN model that has the capability to predict drug release would reduce the workload greatly in the pre-prescription stage, and the adequacy of the predictions has been confirmed [21,35,45,66,74,79,82,86,87,88,89,90,91,92,93,94].

**Figure 5:**
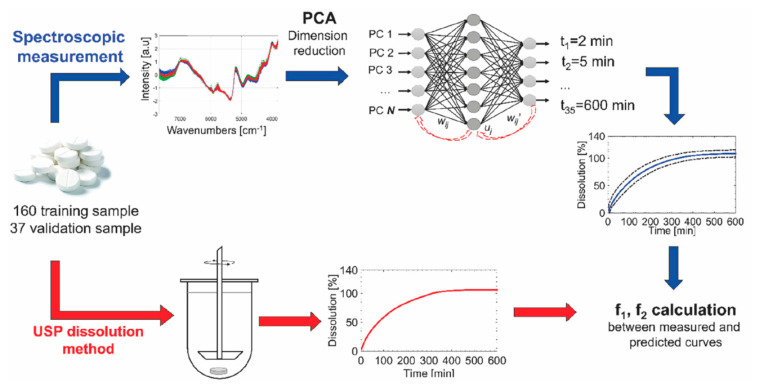
A workflow of the ANN modeling for the prediction of drug dissolution. Reproduced with permission from [96], Elsevier, 2019.

In the case of drug release models, the input variables of the ANNs are the formulation characteristics, such as drug content, pH or composition, while the output neurons represent the dissolution performances of the formulation [95]. Additionally, $f_{2}$ is usually used to evaluate the similarity of drug dissolution profiles. Nagy et al. [96] compared four three-layer ANN models to the traditional PLS regression to predict the dissolution profile of extended release anhydrous caffeine tablets using the NIR and Raman spectra. To be specific, as depicted in Figure 5, the NIR and Raman spectra of each tablet and the experimental dissolution data using the paddle method were measured. The preprocessed spectral data from the principal component analysis (to reduce its dimensionality) were used as the ANN inputs. The result showed that the ANN expressed a lower RMSE of prediction than the PLS, and the reflection NIR spectra ANN model achieved the highest mean $f_{2}$ with the experiment-observed dissolution curve. Hence, compared with other methods, ANNs can figure out the complex relationship between the formulation features and in vitro behavior.

Obeid et al. [97] established an ANN that could show the influence of key process variables on drug dissolution profiles. The optimal ANN model was an MLP (2-3-5) using the BP algorithm, while the input layer neurons were infill density and SA/V ratio and the output layer neurons were the cumulative dissolution of diazepam after 0.5, 1, 2, 4 and 8 h. After training, the $f_{2}$ between the ANN predicted and actual observed values were 70.24 and 77.44 for the two formulations. These results indicated that the ANN could predict the diazepam dissolution of 3D printed tablets. Brahima et al. [98] used an MLP (3-30-1) for the modeling of riboflavin release behavior from poly(NIPA-co-AAc) hydrogels and used the LM algorithm for adjusting the weights. The three factors of time, pH and temperature were the input variables. Meanwhile, 70% of the data (549 release points) was randomly picked for training the network, and the remaining 30% of the data (235 release points) was used for testing. The result showed that the function of the ANN was validated and, compared to that of the response surface methodology (RSM) using the mean square error (MSE) (7.5081 × 10^−4^ and 0.0179, respectively), it was shown that the ANN was more appropriate for the prediction of the release of riboflavin hydrogels and had great generality over the release behavior of the hydrogels.

**Figure 6:**
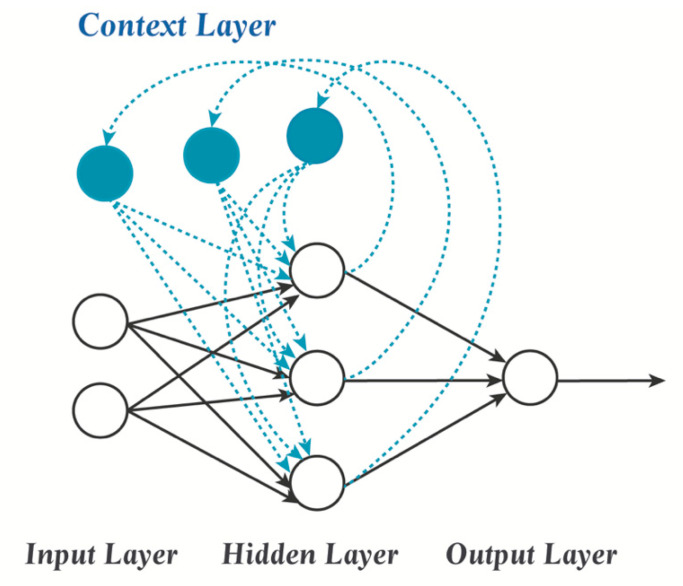
The topology of an Elman neural network. This ENN model input consists of two neurons, while the hidden layer has three neurons and the output layer is composed of one response variable. Among them, each hidden layer neuron has a corresponding memory unit, which will save the state of hidden layer at t. At (t + 1), the neural network will transmit the content of the memory unit and the output of the input layer together to the hidden layer.

In addition, some dynamic neural networks, such as Elman neural networks (ENNs), can be applied to predict drug dissolution profiles [99,100,101]. As shown in Figure 6, an ENN, a typical recurrent neural network, uses recurrent links to provide a dynamic memory and has local feedback. That means that an ENN has a context layer, which is used as temporary memory neurons for the hidden layer output [22,102]. During the training, the hidden layer receives signals from both the input and context layers, and any signal recurrences are regarded as time delays. Since the percentage of the drug to be released at a specific time point can be regarded as time-dependent, it can therefore model drug release behaviors appropriately [103].

Petrović et al. [103] used both an ENN (4-19-10 and the context layer had 18 neurons) and an MLP (4-6-10) to describe the release curves of tablets and demonstrated the wide applicability of ANNs, regardless of tablet types (hydrophilic/lipid). Meanwhile, the genetic algorithm (GA) was used to optimize the number of the hidden layer neurons, weights and signal delays for the ENN. The outcome indicated that the ENN was more suitable for the prediction of dissolution profiles from both hydrophilic matrix tablets ($f_{2}$ were 78.19 and 74.31, respectively) and lipid matrix tablets ($f_{2}$ were 86.91 and 71.34, respectively).

In some studies, ANNs were proposed to optimize the ultrasonic release of APIs in preparations (such as liposomes and micelles) to maintain a constant therapeutic concentration at specific sites [104,105]. For immediate release dosage forms, the disintegrating time of disintegrating oral tablets could also be predicted [49].

Therefore, it is feasible to determine drug release behavior using ANNs according to critical formulation factors, process parameters and API properties. To some degree, ANNs have shown better prediction capabilities compared to others. Furthermore, ANNs can not only accurately predict release properties from formulations but should also be highlighted as a data analysis tool for product quality control in the process of experimentation and production.

### 3.2. Selection and Optimization of Formulations

Formulation development involves a vast number of variable factors, which makes it hard to select and design formulations with APIs of different natures and leads to the need for time-consuming trial and error experiments and complex statistical analysis. Where the critical material properties (CMAs) have been changed, the formulation may need to be adjusted accordingly. Indeed, this is an overloaded and laborious task. Amasya et al. [106] optimized 5-Fluorouracil (5-FU) lipid nanoparticle formulation using an ANN. The CMAs (types of triglycerides, amount of solid lipid, the presence of co-lipid, the presence of liquid lipid and the drug concentrations) of the lipid nanoparticles were considered as the inputs and the 5-FU lipid nanoparticle characterizations were considered as the outputs. The selected formulation by the ANN had the desired physicopharmaceutical attributes, efficacy and skin permeation efficiency for dermal drug administration, so ANNs can obtain high quality formulations and save time and cost.

Due to their simplicity and easy implementation, some computer-based design of experiments (DoE) methods have been proposed. RSMs have a high sensitivity to independent variable composition and constitute one of the most widely used methods [78,107,108]. On one hand, an RSM is based on first-, second- or third-order low dimensional polynomial equations [109], leading to its lack of fitting and optimization abilities for a multiple response model based on limited variables. On the other hand, DoEs require a prior knowledge of the manufacturing process and need to experiment based on the process parameters proposed by the software in order to support the establishment of the model [97,110,111]. In comparison, ANNs have the reliable ability to find relationships between various factors, which can cover the relationship between all inputs and outputs with a neural network graph. In a DoE experiment, each dependent variable requires a separate model [109] and ANNs are capable of ranking the contributions of the materials and processing factors (input layer parameters) that affect the key properties of the formulations. Therefore, many ANN models can be used to conduct fundamental research of formulation and preparation techniques, which demonstrates the superiority of ANNs [51,60,63,64,68,70,100,112,113,114,115,116,117,118].

Koletti et al. [119] compared two preparation methods (nanoprecipitation and two-step desolvation) of diclofenac sodium gelatin nanoparticles using two optimization techniques (multilinear regression and an ANN). It was proved by the experiment that the ANN with the standard BP algorithm showed a significantly improved ability for the prediction of particle size, ζ-potential and encapsulation efficiency (EE) compared to the multilinear regression. Li et al. [109] optimized verapamil hydrochloride polymer–lipid hybrid nanoparticles (PLN) formulation using an RSM and ANN, and the prediction performance of the RSM and ANN were compared. The ANN structures were 3-2-1 for analyzing EE and 3-3-1 for analyzing mean particle size. They found that the PLN guided by the ANN showed a high EE (92.4%) and desirable mean particle size (~100 nm). ANNs were an effective approach to optimize PLN formulations with a stronger recognition and prediction ability. Reza Zaki et al. [78] employed an RSM and ANN combined with the GA (optimizing the number of hidden neurons) to improve and simulate the manufacturing process of agar nanospheres. Compared to the RSM, the ANN proposed optimum data closer to the desired values for all responses, except PDI. Thus, ANN techniques are suggested for formulation optimization.

Considering CMAs and critical process parameters, Sansare et al. [120] used two types of BP neural networks to improve the liposomes preparation process: a multi-input multi-output (MIMO) and multi-input single-output (MISO) model. For the MIMO model, an ANN was used to construct all relationships. In the MISO model, the influence of five features on one dependent variable (particle size or PDI) was considered at a time, hence the weights and thresholds of each output were different. They used the Levenberg–Marquardt (LM) optimization algorithm and the tangent sigmoid activation function. In a set with 175 data, the mean relative error of the particle size and PDI of the MISO were lower than the MIMO. Meanwhile, the addition of molecular descriptors (as input parameters) could significantly reduce prediction errors. Therefore, ANNs can assist in the design of formulations.

**Figure 7:**
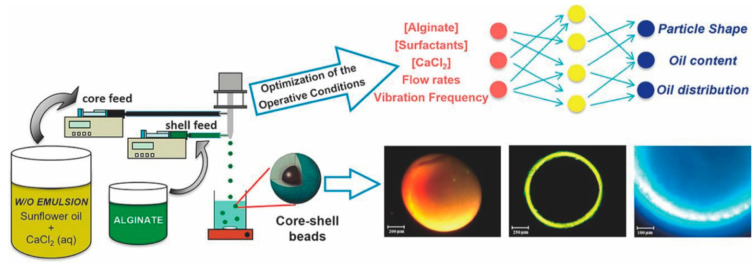
The formulation optimization process of core-shell microparticles in which an ANN participated. Reproduced with permission from [121], Elsevier, 2018.

The microencapsulation of hydrophobic API with a hydrophilic matrix can improve the stability of a drug. As shown in Figure 7, Rodríguez-Dorado et al. [121] prepared alginate core-shell microparticles by drawing a water-in-oil emulsion and an alginate solution into each syringe, pumping them into the coaxial nozzle and then extruding. To select the optimal operating conditions, they implemented an MLP (7-4-3) combined with the GA. Seven process parameters were introduced as the input variables: the alginate percentage; the concentration of CaCl_2_ in the emulsion; the concentration of Tween 85 in the emulsion; the concentration of Tween 85 in the receptor bath; the alginate flow rates; the emulsion flow rates; and the vibration frequency. Meanwhile, 39 pieces of data were used for training and 4 for testing the error. The optimized core-shell microparticles formulation by the MLP successfully obtained a narrow size distribution and uniform oil distribution in the particle core and it had the highest mechanical resistance to maintain the stability of the microparticles. The results demonstrated that the use of an ANN facilitated the understanding and optimization of complex manufacturing processes through reducing preliminary experiments.

In general, where the relationship between the independent and dependent variables is straightforward and of a lower dimensionality, response surface methodology and artificial neural networks can both present valuable outcomes [22,108]. However, ANNs possess more fitting properties than RSMs because they can adjust the number of neurons to improve the availability flexibly if the unknown pattern is intricate.

Due to the complex nature of formulation development, ANNs have certain uses for modeling and analyzing the process of formulation selection and optimization, which can greatly lighten the workload and reduce the required time. By and large, ANNs have better prospects as valuable pre-formulation tools and they are also suitable for the optimization of some manufacturing process parameters [34,122,123].

### 3.3. Establishment of In Vitro–In Vivo Correlation

In recent years, drug candidates have increased substantially and the number of clinical studies has doubled, resulting in high development and supervision costs. Therefore, ethical reasons and the purpose of more effective drug research and supervision promote the establishment of validated IVIVC. The primary aim of IVIVC is to predict drug plasma concentration–time profiles by relating the drug dissolution behavior in vitro (usually the dissolution rate or degree) to the pharmacokinetic process in vivo [124,125,126]. It can reduce the workload of each phase while characterizing the product performance and can provide guidance and support for drug development (such as the reduction in animal experiments), production changes (such as the replacement of bioequivalence) and supervision and management (such as the quality standard establishment of dissolution rate). Many IVIVC models use a linear approach. We know that ANNs applied to an IVIVC have additional advantages over some classical regression approaches in data analysis [36,58,72,73,81,127,128,129]. Therefore, the IVIVC could be established by an ANN.

G. Fatouros et al. [127] introduced an ANN model with neuro-fuzzy to establish an IVIVC (AFM-IVIVC) of probucol self-emulsifying DDS. The in vitro experiment, based on the lipolysis model, was performed under agitation at 37 °C and the pH was kept at 6.5. The determinations of the drug plasma concentration were sampled from pigs at 12 time points: 0, 0.75, 1.5, 2.25, 3, 4, 5, 6, 8, 12, 24 and 48 h. The predicted mean plasma concentration by AFM-IVIVC were closer to the data observed in vivo. These preliminary results suggested that the abilities of an ANN for determining IVIVC have been demonstrated and the potentials for predicting drug behavior in vivo have been offered.

At the same time, an ANN can also be applied to pharmacokinetic study [3,130,131,132,133]. For instance, the estimation of some parameters (such as the apparent volume of distribution and the total clearance) in humans based on animal data using an ANN is feasible [3,133,134]. Iwata et al. [133] selected the chemical structure of the drug (the core tensor selected by a multimodal deep tensor model) and rat clearance data as the two variables of the input layer and they successfully developed a new human drug clearance prediction method using an ANN.

Although PLS regression analysis indicates the simplest behavior, it is intelligible that IVIVC should not be limited to a linear relationship [73]. An IVIVC built by an ANN showed efficient prediction performance as well as the potential to gain complex relationships and pharmacokinetic parameters. It is conceivable that ANNs can advance the application and progress of in vitro–in vivo correlation.

### 3.4. Other Applications of Artificial Neural Network

In addition to the applications mentioned above, ANNs can be used to determine drug concentration [76,80], predict transdermal permeability [135,136], determine the critical quality attributes that affect a certain formulation property [75,137,138,139,140], predict the intrinsic solubility of drugs [141,142], control the granulation process within a fluidized bed [50], predict the stability of dosage forms [143,144,145], characterize physicochemical properties [146], control drug quality in multicomponent formulation with overlapping spectra [65,147] and predict complex colloidal delivery systems phase behavior [37,148,149,150,151] so as to improve the efficiency of conducting various related processes.

In a recent study [152], an ANN (3-7-3) in conjunction with the GA was established to analyze the correlation between hot melt extrusion process parameters and dapivirine vaginal film performance. In another study, Lou et al. [153] explored the relationship between raw material attribute inputs and product profile outputs (tensile strength and brittleness index) for tablets using a core/shell technique. They selected six analysis tools: an RSM; a support vector regression; and four ANN models (BPNN, GA-based BPNN, mind evolutionary algorithm-based BPNN and an extreme learning machine). Compared to others, the four ANN models provided better prediction abilities for tensile strength and brittleness index. Vidoviča et al. [154] used an ANN to predict the API dissolution in 30 min (Q30) and to determine the more critical quality attributes impacting the API dissolution behavior. The optimal ANN model (75-1-3-1) used Tanh as the activation function. They found that the API particle size had the most impact on Q30 by investigating the Q30 susceptibility to changes of 10% in the value of the independent variables.

Damiati et al. [155] utilized an ANN to predict the effects of appropriate hydrotropic agents on a poorly water-soluble drug’s apparent aqueous solubility. They built the ANN (10-2-2-1) and employed the time-invariant noise algorithm to avoid being caught in the local minimum value. The validated ANN produced highly accurate predictions of the indomethacin solubility of hydrotropes at different concentrations (R^2^ = 0.998). The results showed that a more general ANN was developed and could predict drug solubility for a range of hydrotropic systems. In another study, Chiappini et al. used an MLP to explore the relationship between fluorescent signals and etanercept concentration (the dependent variable of the output layer) [156]. The number of neurons in the input layer and the two hidden layers of the best obtained MLP topology were 12, 2 and 0, respectively. The analyzed results illustrated that the linear relationship between the fluorescence signals and etanercept concentration was very poor and that the prediction quality of the best structure MLP model was better than the partial least squares (PLS) and the mean relative error, about 7.0% and 12.6%, respectively, which meant that the established optimal ANN model provided a faster etanercept concentration detection method.

For microparticle formulations, the problems of needle blockages and microparticle residue caused by the micron level size and a faster sedimentation rate during injection is still a challenge today. Sarmadi et al. [157] used an ANN (2-10-1) to predict the injectability of microparticles by understanding the key parameters (particle size, needle size and solution viscosity) affecting the transport of the microparticles and designing an injection device to improve the delivery of the drugs in subcutaneous administration. A BP with the LM algorithm was applied to train the network and 319 data points were selected as the inputs. There was a robust correlation between the predicted and the actual injectability (R^2^ = 0.90 for the testing data).

For implants, a cumulative drug concentration is hardly detected at the implantation site because with the extrinsic/intrinsic hydrodynamic conditions, some factors also play a vital role in drug release in addition to formulation factors. In one study, researchers used an ANN to clarify the physicodynamic phenomenon controlling the diffusion coefficient of a sodium dodecyl sulphate (SDS) gel and to find the greatest influential factor on the diffusivity from formulation variables (SDS loading dose and gel weight), intrinsic variables (flow rate and pH of vagina fluids) and extrinsic variables (application site) [137]. The results illustrated that the initial burst release phase could be controlled by the drug loading and preparation weight; however, the polymer hydrolytic erosion was the chief mechanism. Among the five inputs, the pH of the vagina fluids was the leading factor affecting the gel release behavior.

As an important property of a solid dosage form, porosity can indirectly characterize its form dissolution capability and rate. Khalid et al. [53] selected four computational intelligence models (decision trees, random forests, an ANN and symbolic regression) to predict the porosity of tablets. Among them, the ANN was the best performing method (NRMSE = 1%). Meanwhile, ANNs also have the ability to identify factors affecting the properties of sustained or controlled release DDSs, such as microspheres, nanoemulsions, nanoparticles, micelles and controlled release tablets [67,121,138,158,159,160]. In another study, the benefits of using a deep learning method to consider a time series feature when studying drug delivery using high-content imaging were also highlighted [161].

Additionally, 3D printing, an automated and digitalized technology, has exceptional advantages in many fields and is also used in the pharmaceutics industry to drive personalized medicine [162]. With 3D printing technology, preparations can be produced directly from a computer-aided design model using layer-by-layer manners, such as fused deposition modeling, direct powder extrusion and powder binding. An ANN can also be applied to optimize the process parameters, inspect quality and inspire innovative dosage-form design in 3D-printed drugs [30,111,162,163].

The particle or granule size (distribution) is a critical quality attribute for drug dosage forms and even subtle differences may generate significant impacts. ANNs provide a useful tool for the prediction of particle/granule size [7,91,164,165,166]. Shirazian et al. [91] trained an ANN to predict the granule size distribution (D_10_, D_50_ and D_90_) of wet granulation. The best model had two hidden layers containing two neurons per layer. The ANN produced a high correlation level between the experimental and predicted data for the size distribution (R^2^ > 0.99). Therefore, the developed model could offer accurate predictions for the twin-screw granulation process in this study. Damiati et al. [166] developed individual ANN models for three microfluidic technologies, as well as joint general ANN models comprising two and three devices to predict the size of PLGA microparticles produced by microfluidic systems. The results showed that not only can the one-to-one ANN models provide accurate microparticle size prediction (R^2^ > 0.98 for all validation datasets), but the joint ANNs comprising two and three microfluidic devices can also predict the size of droplets and microparticles with a high degree of accuracy (R^2^ for validation dataset equaled 0.948 and 0.971, respectively). In another research [167], an ANN was also applied to estimate the effects of particle size and wall shear rate on the number of nanoparticles adhering to the vessel walls and it proved that there was an optimal particle size to maximize the number of adhered particles.

Youshia et al. [164] predicted the particle size of polymer nanoparticles using an ANN. The ANN was trained by the BP algorithm and the hyperbolic tangent was the activation function. The acquired model provided reliable prediction performances for the size of nanoparticles prepared by some new polymers in the range of 70~400 nm. They found that the polymer surface activity (PVA concentration) had the greatest effect on particle size, followed by the molecular weight and the hydrophobicity. It showed that the intelligent system for the prediction of the size of the nanoparticles using an ANN was an effective way to improve the efficiency and accuracy of nanoparticles development.

Drug stability is the main research content of quality control, which runs through the whole process of formulation research and development, such as the guidance of formulation design, the determination of storage conditions and validity term. Ajdarić et al. [143] used a four-layer MLP to predict the stability profile of esomeprazole and to determine the pH range for the reconstituted solution of freeze-dried formulations to ensure product quality over the course of the shelf life. The input layer consisted of two variables: the pH value of the esomeprazole solution and the period of storage. The content of the esomeprazole and four main impurities were the dependent variables of the ANN. Moreover, it took at least three to six months to conduct traditional stability experiments, which is time-consuming and produced an inadequate predictability. Han et al. [144] collected 646 stability data points and used 8 ML methods, such as ANN, random forests and decision trees, to establish a prediction model for the solid dispersion stability for 3 months and 6 months (“1” for stable and “0” for unstable). Among them, the ANN had the better drug stability prediction accuracy for 3 months and 6 months in the testing set, which were 84.17% and 82.50%, respectively. Gentiluomo et al. [145] predicted the 6-month long-term stability of therapeutic protein formulations under three storage temperatures (at 4 °C, 25 °C and 40 °C) based on accelerated stability studies. In total, 11 Bayesian regularization BP neural network models with the LM optimization algorithm were constructed and a cross-validated method in the test and validation sets was used to determine the generalization ability. They found that the ANN provided accurate characterizations and predictions of the protein stability under different storage conditions.

The applications of ANNs in pharmaceutical science are broad, ranging from the design of dosage forms and characterization of formulation to the prediction of API release, the interpretation of analyzed data and the establishment of IVIVC, etc. Based on these findings, ANNs have been applied to evaluate the relationship between data and to guide the traditional experiments, which could minimize the number of required experiments. Hence, it has practical guidance and optimization significance for pharmaceutical sciences.

## 4. Conclusions and Perspectives

Alan Turing introduced the concept of a machine that could think in 1950. “Artificial intelligence” as a term was proposed by John McCarthy in 1956, and AI began to take shape. In recent years, the popularity and practicability of AI have been developed tremendously. With their powerful functions, artificial neural networks (ANNs) and other artificial intelligence technologies have gradually penetrated into many aspects of pharmaceutical science. Among these, the ANN is the most frequently applied modeling technique.

An artificial neural network is the integration and development of various methods, which has the characteristics of comprehensiveness and interdisciplinarity. Although current pharmaceutical development still mainly relies on the traditional trial and error methods of pharmaceutical researchers, we believe that the integrated experimental, theoretical and task-driven/data-driven AI methodology will vastly promote the betterment of pharmaceutical science. It is clear that the communication between ANNs and pharmaceutics can avoid repetitive work, such as greatly reducing the demand for animal research, predicting drug pharmacokinetic profiles, optimizing formulation designs, simplifying quality control, accelerating consistency evaluations, etc. Thus, the time and cost of fundamental research and product development can be reduced and the burden of drug evaluation and supervision could be decreased. Moreover, ANNs can generalize correlation tendencies using limited input data so as to carry out specific tasks quickly and efficiently. In short, ANNs have the possibility of becoming a key step in the research process of pharmaceutics and the associated data mining by ANNs could provide us with the capacity to predict outcomes and discover new relationships.

The application of ANNs in pharmaceutical formulation emphasizes the importance of model selection and construction. In general, the successful establishment of ANN-based models is measured by its predictive ability, which is dependent on the selection of inputs, the time spent on training and the construction of the ANN architecture. Thus, before constructing an ANN model, some theoretical knowledge is required and the whole ANN framework needs to be considered seriously. Additionally, a universal learning method, optimization algorithm or network configuration are impossible for ANN application in pharmaceutics, and many attempts are required to choose an appropriate network structure when facing a new problem. The ultimate goal of introducing ANNs into pharmaceutical science is to solve problems in practical applications, so some factors, such as running time and running memory, should be considered to create ANN models of more practical value.

For the specific applications of ANNs in pharmaceutical formulation, some traditional release models are not applicable in all cases and ANNs based on ML have good data processing capabilities, so ANNs are expected to be advantageous in the establishment of drug release models. We think that ANNs will be used in the study of drug release mechanisms in the human environment when more physicochemical and physiological factors are taken into consideration.

We also hope that ANNs can promote drug industrialization and be applied to preparation production and analysis. In recent years, pharmaceutical companies have been encouraged to adopt continuous manufacturing technology, which demands product understanding, process understanding and process control based strongly on the concept of Quality by Design. The various superiorities of ANNs can contribute to the design of better formulations and the development of more robust manufacturing processes. At the same time, process analysis technology has also begun to be proposed to help confirm the product quality in real time. It is conceivable that, to some extent, it is feasible to predict the CQAs of products based on ANNs for quality assurance purposes. These worthy attempts are expected to have a real positive impact on the pharmaceutical industry.

Although ANNs are expected to be a powerful tool in pharmaceutical research, it does not mean that some conventional experimental methods should be abandoned. The superiorities of conventional and emerging techniques should be integrated. In addition, it is feasible to extend the application of ANNs to the problems of the prediction, characterization and optimization of pharmaceutical formulation only on the basis of understanding the prescription ingredients, processing parameters and target properties. However, there are some limitations for ANN applications. For example, the amount of workable data in pharmaceutics is too small compared to the enormous amount of information available in natural language processing, image processing, etc. and open data sharing in the field of pharmaceutics is not common. Moreover, the accuracy of ANNs in drug and formulation analysis ought to be improved and new modeling tools also need to be raised. In the future, advances in optimization algorithms, model design and upstream disciplines will further improve the performance of ANNs, and then more potential applications of ANNs in pharmaceutics can be explored.

At present, the ANNs commonly used in pharmaceutical science mainly includes the aforementioned multilayer perceptron, generalized regression neural network, etc. In the future, we should conduct more in-depth explorations of ANNs and continue to mine their value in our research field. It can also be said that we should use ML and AL technologies in the field of pharmaceutics more widely, so as to encourage the communication among these disciplines and create more innovation and breakthrough sparks.

## Figures and Tables

**Table 1:** The latest applications of ANNs in pharmaceutical science.

Neural Network	Training Algorithm	Optimization Algorithm	Architecture (Input–Hidden–Output Layer)	Application	Reference
MLP	BP	BFGS	2 input variables, 1 output variable	To assess the stability of meropenem in human plasma at −20 °C.	[42]
MLP	BP	N/A	4-4-3	To investigate the correlation of various process variables affecting the properties of albumin-loaded chitosan nanoparticles.	[43]
MLP	BP	/(best)	101-7-1, 101-10-1	To quantitatively analyze amoxicillin and flucloxacillin in the binary mixtures.	[44]
MLP	BP	BR (best)	3-5-53	To predict the dissolution curve of extended release drotaverine tablets.	[45]
MLP	BP	SCG	15-5-1, 11-8-1	To predict the apparent degree of supersaturation in two supersaturated lipid-based formulations.	[46]
GRNN	N/A	N/A	7-45-6-5	To determine the key properties affecting granule size in the fluidized bed granulation process and to predict granule characteristics.	[47]
MLP	BP	LM	2-10-1	To predict turbidity for determining particle size and the stability of emulsions.	[48]
MLP	BP	GD	3-4-1	To predict the disintegration time of disintegrating oral tablets.	[49]
GRNN	K-means	N/A	2-9-5-4	To predict the drug stability and shelf life of aspirin tablets at different storage temperatures (30 °C, 40 °C, 50 °C and 60 °C).	[32]
MLP	BP	GD	4-12-12-1	To predict the temperature distribution of the fluidized bed for controlling the granulation step.	[50]
MLP	Resilient BP	N/A	3-3-3	To optimize ketoprofen solid lipid nanoparticles gel for topical delivery.	[51]
MLP	BP	N/A	11-8-6-5	To describe PLGA microsphere release profiles.	[52]
MLP	BP	N/A	3-13-11-1	To predict whether tablet porosity is composed of microcrystalline cellulose and lactose.	[53]
MLP	BP	GA	4-11-1	To predict the particle size of the nanoemulsion system and to investigate the factors influencing particle size.	[54]
GRNN	K-means	N/A	2-10-6-5	To optimize the drug release behavior of extended release diclofenac sodium pellets in vitro.	[35]
MLP	BP	GA	3-4-1	To determinate fluoxetine concentration using the UV spectrophotometric method.	[55]
MLP	BP	LM	3-10-1	To improve the key parameters affecting the size of a self-emulsifying drug delivery system (DDS).	[56]
MLP	BP	BR	(4-103)-6-53 (best)	To predict the release profile of sustained release tablets in real time.	[57]
MLP	BP	Step rule	2-6-2	To establish the IVIVC for osmotic release nifedipine tablets.	[58]
GRNN	K-means	N/A	2-27-2-1	To predict the microemulsion phase boundaries in the quaternary system.	[37]
MLP	BP	LM	3-10-4	To optimize the HPLC method to simultaneously analyze cyclosporin A and etodolac in solution, human plasma, nanocapsules and emulsions.	[59]
MLP	BP	N/A	3-5-3	To select terbutaline sulfate nanogel formulation for transdermal delivery.	[60]
DNN	BP	BGD	10 layers (50 hidden neurons on each layer), 9 layers (30 hidden neurons on each layer)	To predict the dissolution/release characteristics of two formulations (fast disintegrating films and sustained release matrix tablets).	[19]
MLP	BP	N/A	2-3-1	To capture the effects of gelatin and cholesterol incorporation in the sodium salicylate liposomes on EE.	[61]
MLP	BP	GD	9-50-50-50-50-1 (best)	To predict the impact of the structure and properties of inhaled dry powder components on fine particle fraction.	[62]
MLP	BP	N/A	4-8-8	To optimize doxorubicin amphiphilic polymeric nanoformulations.	[63]
MLP	BP	BFGS	3-3-7 (best)	To optimize lamotrigine hydrogel formulation.	[64]
MLP	BP	/ and GA	181-7-1, 181-10-1 and 72-3-1, 36-3-1	To quantitatively analyze velpatasvir and sofosbuvir in the binary mixture.	[65]
MLP	BP	SCGD	2-4-2	To study the impact of aprepitant liquisolid formulation variables on dissolution performances.	[66]
MLP	BP	N/A	3-5-2	To investigate the effects of changes in nanoemulsion formulation on stability and cells viability.	[67]
MLP	BP	GA	3-9-1	To optimize ophthalmic pilocarpine hydrochloride flexible nano-liposomes.	[68]
MLP	BP	N/A	2-20-10-1	To select the crucial variables affecting drug dissolution in the solid lipid extrudates.	[69]
MLP	PSO (best)	N/A	7-4-8	To develop mini-tablets preformulation.	[70]
MLP	BP	LM	3-7-1	To determinate the parameters controlling sodium tripolyphosphate nanoparticles size and yield.	[71]
MLP	BP	N/A	3-2-1	To model the IVIVC for inhaled salbutamol administered via nebulizer.	[72]
GRNN	N/A	N/A	8-8-16-15	To model the IVIVC for a sustained release paracetamol matrix tablet.	[73]
GRNN	K-means	N/A	2-10-7-6	To predict the release behavior of extended release aspirin tablets in vitro	[21]
MLP	BP	BFGS	4-6-6	To evaluate the influence of various factors on the release behavior of a prednisone multiple-unit pellet system.	[74]
MLP	BP	N/A	3-1-1	To determine the key elements influencing the particle size of mebudipine nanoemulsion.	[75]
MLP	BP	LM (best)	N/A	To predict the concentrations of emtricitabine and tenofovir alafenamide fumarate using a spectrophotometry technique.	[76]
Monmlp, DNN	N/A	LASSO	N/A	To screen the critical quality attributes of PLGA and minimize the prediction error of PLGA microspheres release profiles.	[77]
MLP	BP	GA	4 input variables, 5 output variables	To optimize the manufacturing process of Bupropione HCl-loaded agar nanospheres.	[78]
MLP	BP	SCGD	4-4-5, 4-4-3	To predict the swelling and erosion steps of nimodipine hydrophilic matrix tablets using a conventional model and statistical moment model.	[79]
MLP	BP	LM (best)	106-8-7	To determinate paracetamol and chlorzoxazone concentrations with their five process-related impurities using a UV-spectrophotometer.	[80]

BFGS: Broyden–Fletcher–Goldfarb–Shanno; N/A: not mentioned in the reference; MLP: multilayer perceptron; GRNN: generalized regression neural network; BP: backpropagation; BR: Bayesian regularization; SCG: scaled conjugate gradient; LM: Levenberg–Marquardt; GD: gradient descent; PLGA: poly(lactide-co-glycolide); GA: genetic algorithm; BGD: batch gradient descent; DNN: deep neural network; PSO: particle swarm optimization; IVIVC: in vitro–in vivo correlation; Monmlp: monotonic multilayer perceptron; LASSO: least absolute shrinkage and selection operator; SCGD: scaled conjugate gradient descent; HPLC: high performance liquid chromatography.

## Data Availability

Not Applicable.
